# Older Persons’ Transitions in Care (OPTIC): a study protocol

**DOI:** 10.1186/1471-2318-12-75

**Published:** 2012-12-14

**Authors:** Greta G Cummings, R Colin Reid, Carole A Estabrooks, Peter G Norton, Garnet E Cummings, Brian H Rowe, Stephanie L Abel, Laura Bissell, Joan L Bottorff, Carole A Robinson, Adrian Wagg, Jacques S Lee, Susan L Lynch, Elmabrok Masaoud

**Affiliations:** 1 Faculty of Nursing, University of Alberta, Edmonton, AB, Canada; 2 School of Health and Exercise Sciences, University of British Columbia’s Okanagan campus, Kelowna, BC, Canada; 3 Department of Family Medicine, Faculty of Medicine, University of Calgary, Calgary, AB, Canada; 4 Department of Emergency Medicine, Faculty of Medicine and Dentistry and School of Public Health, University of Alberta, Edmonton, AB, Canada; 5 School of Nursing, University of British Columbia’s Okanagan campus, Kelowna, BC, Canada; 6 Division of Geriatric Medicine, Department of Medicine, Faculty of Medicine and Dentistry, University of Alberta, Edmonton, AB, Canada; 7 Department of Emergency Services, Sunnybrook Health Sciences Center, Toronto, ON, Canada; 8 Faculty of Nursing, Edmonton Clinic Health Academy, University of Alberta, 11405-87 Ave, Edmonton, AB, T6G 0C1, Canada

**Keywords:** Seniors, Elderly, Transitions, Quality of care, Handovers, Communications, Emergency Departments, Emergency Medical Services, Nursing homes, Measurement

## Abstract

**Background:**

Changes in health status, triggered by events such as infections, falls, and geriatric syndromes, are common among nursing home (NH) residents and necessitate transitions between NHs and Emergency Departments (EDs). During transitions, residents frequently experience care that is delayed, unnecessary, not evidence-based, potentially unsafe, and fragmented. Furthermore, a high proportion of residents and their family caregivers report substantial unmet needs during transitions. This study is part of a program of research whose overall aim is to improve quality of care for frail older adults who reside in NHs. The purpose of this study is to identify successful transitions from multiple perspectives and to identify organizational and individual factors related to transition success, in order to inform improvements in care for frail elderly NH residents during transitions to and from acute care. Specific objectives are to:

1. define successful and unsuccessful elements of transitions from multiple perspectives;

2. develop and test a practical tool to assess transition success;

3. assess transition processes in a discrete set of transfers in two study sites over a one year period;

4. assess the influence of organizational factors in key practice locations, e.g., NHs, emergency medical services (EMS), and EDs, on transition success; and

5. identify opportunities for evidence-informed management and quality improvement decisions related to the management of NH – ED transitions.

**Methods/Design:**

This is a mixed-methods observational study incorporating an integrated knowledge translation (IKT) approach. It uses data from multiple levels (facility, care unit, individual) and sources (healthcare providers, residents, health records, and administrative databases).

**Discussion:**

Key to study success is operationalizing the IKT approach by using a partnership model in which the OPTIC governance structure provides for team decision-makers and researchers to participate equally in developing study goals, design, data collection, analysis and implications of findings. As preliminary and ongoing study findings are developed, their implications for practice and policy in study settings will be discussed by the research team and shared with study site administrators and staff. The study is designed to investigate the complexities of transitions and to enhance the potential for successful and sustained improvement of these transitions.

## Background

In 2006, the number of Canadians aged 65 and over exceeded four million for the first time [1]. By 2011, this represented 14.8% of the entire population (11.1% in Alberta; 15.7% in British Columbia – the two provinces represented in the present study) [2]. The greatest increase is among those aged 85 and older, with an average annual rate of growth reported to be 3.8% [3,4]. By 2050, the over 85 age group could account for one-fifth of all older persons [4]. These demographic trends increasingly challenge healthcare systems as older people often require both more and different health services than do younger people [5].

Similar to other Organization for Economic Cooperation & Development (OECD) countries, almost half of Canadian seniors will be residents of nursing homes (NHs) at some point during their lives [6-8]. Almost half (45%) of Canadians in NHs are 80+ years of age, over half suffer from dementia [9-11] and a substantial majority (73%) are women [6]. NH residents form a highly vulnerable group with complex care needs and a high degree of dependency on care providers [12].

Changes in health status among NH residents – often triggered by events such as falls [13], infection [14], depression and other changes in mental status [15], and failure to thrive [16] – necessitate transitions between NHs and emergency department (ED). While research has shown that not all emergency transitions are required, transferred residents frequently experience care that is unnecessary, delayed, not evidence-based, potentially unsafe, and fragmented [17-21]. Furthermore, a high proportion of residents and their family caregivers report substantial unmet needs resulting from transitions [22,23].

### Study purpose & objectives

The Older Persons’ Transitions in Care (OPTIC) study is a three year Canadian Institutes of Health Research Partnership for Health System Improvement (CIHR-PHSI) grant that involves the Central Okanagan district of the Interior Health (IH) region in British Columbia and the Edmonton Zone of Alberta Health Services (AHS) in Alberta. The purpose of this study is to identify successful transitions from multiple perspectives and to identify organizational and individual factors related to transition success, in order to inform improvements in care for frail elderly NH residents during transitions to and from acute care. Specific objectives are to:

1. 1.define successful and unsuccessful elements of transitions from multiple perspectives;

2. 2.develop and test a practical tool to assess transition success;

3. 3.assess transition processes in a discrete set of transfers in two study sites over a one year period;

4. assess the influence of organizational factors in key practice locations, e.g., NHs, emergency medical services (EMS), and EDs, on transition success; and

5. identify opportunities for evidence-informed management and quality improvement decisions related to the management of NH – ED transitions.

### Rationale

While a number of US investigators have reported on transitions for NH residents, in particular NH-ED transitions [24-29], few Canadian studies exist that report on transitions for NH residents [30-32]. We do know that the in-hospital, and in particular ED experience for the frail NH resident is characterized by serious quality and safety concerns [33-36]. Sub-optimal quality of care in NH settings [37], and in pre-hospital and ED settings [38,39] have been described in many international [8], national [40], and provincial reports [41]. These reports also highlight high rates of burnout among family caregivers [39,42]. Given these reports and the cognitive impairment of many NH residents [9-11], the complexity of delivering effective and appropriate care can increase during transitions [36,43]. This complexity is often made more difficult by compromised communication between different agencies involved in the transition [44].

Previous research linking organizational factors with the success or quality of the transition process signals the need for further exploration. Carter linked 1991–1993 Medicaid reimbursement data from Massachusetts with specific facility-level organizational and structural attributes and showed that nursing home case-mix and local hospital bed supply levels predicted hospitalization rates of NH residents [45]. However, Boockvar and Burack found no management level relationships between NHs, hospitals and the quality of transitions [26].

Little attention has been devoted to developing measures that could address the quality of care delivered and received during transitions from NH to ED. One instrument, Coleman’s *Care Transitions Measure* tool, has been used to measure performance around transitions [28,29,46,47], primarily focusing on identifying care deficiencies and approaches to address these deficiencies. However, this is a self-report tool which has been used only with community based participants and is therefore not appropriate for most residents in NHs, who have some degree of cognitive impairment [9-11]. Saliba and associates developed a structured implicit review form for use with retrospective chart audits to determine the appropriateness of ED transitions [48]. This tool is useful for research purposes; however, it is less applicable to the needs of decision-makers and managers who need data for quality management prospectively. Members of our research team have previously constructed instruments to measure contextual factors in nursing homes that influence care outcomes [49]. In that work we established two criteria for such a measure: (1) feasibility (brevity and ease of completion; the instrument can be completed in 10–15 minutes), and (2) modifiability (focus on concepts that are potentially modifiable). We will apply these criteria to the development of the transitions success tool.

## Methods

### Design

This is a mixed-methods observational study using data from multiple levels (facility, care unit, individual) and sources (healthcare providers, residents, residents’ families, health records, and administrative databases). We will examine the quality of transitions of NH residents between and among three care settings (NHs, EMS, and EDs) over a one-year period in two cities in the provinces of British Columbia and Alberta. The study’s governance structure is founded on an integrated knowledge translation (IKT) approach. The CIHR-PHSI granting vehicle provides for decision-makers and researchers on the OPTIC team to participate equally in developing the study goals, design, data collection, analysis, recommendations and dissemination of findings. As preliminary and ongoing study findings are gathered and interpreted, their implications for practice and policy in study settings will be discussed and shared with study site administrators and staff, rather than waiting for traditional modes of academic dissemination of findings before changes to the workplace are made.

### Theoretical framing of the study

**Figure 1:**
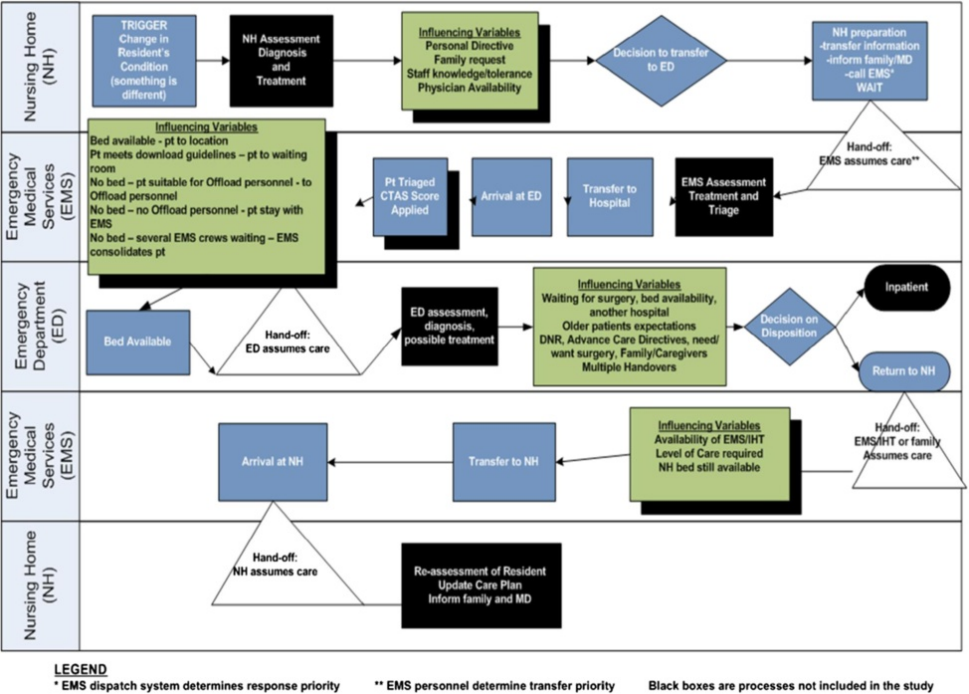
**OPTIC Transitions Framework.** Developed by the OPTIC Team from Parke & Hunter, 2009.

**Figure 2:**
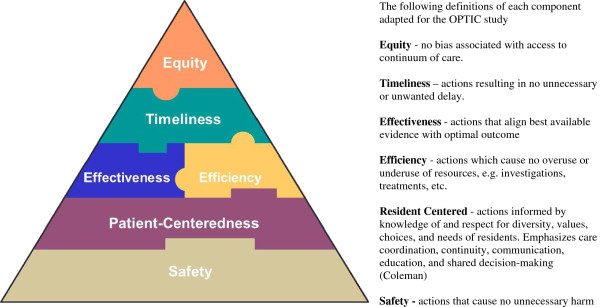
The Institute of Medicine Model for Quality in Healthcare.

The research team developed the OPTIC Transition Framework (Figure 1) informed by prior work of Parke and Hunter [50] to guide data collection procedures. Our work is also informed by the Institute of Medicine (IOM) quality framework [51] (Figure 2). The IOM model stresses that health care and its systems and processes should be *safe* – avoiding injuries to patients from the care that is intended to help them; *effective* – providing services based on scientific knowledge to all who could benefit, and refraining from providing services to those not likely to benefit; *patientcentred* – providing care that is respectful of and responsive to individual patient preferences, needs, and values, and ensuring that patients’ values guide all clinical decisions; *timely* – reducing waits and sometimes harmful delays for both those who receive and those who provide care; *efficient* – avoiding waste, including that of equipment, supplies, ideas, and energy; and, *equitable* – providing care that does not vary in quality because of personal characteristics such as age, gender, ethnicity, geographic location, and socioeconomic status. Thus our work will identify and propose system and process improvements that address several of these six elements of a quality healthcare system [51].

**Figure 3:**
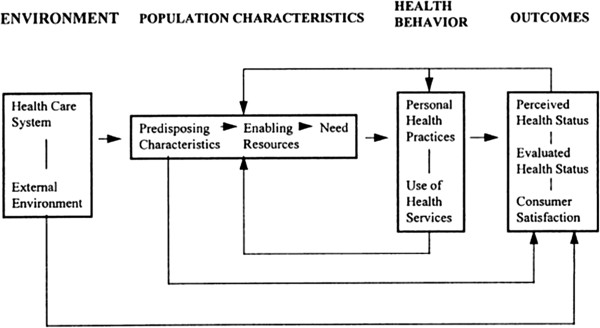
Andersen Behavioral Model of Health Services Use.

The design and analysis of questions related to factors that influence the success of implementing new models of care is guided by the Andersen Behavioral Model of Health Services Use [52] shown in Figure 3. The Andersen model specifies relationships among contextual factors (e.g., environmental, population, health behaviour, and outcomes) and population characteristics (e.g., need, access, and predisposing characteristics) that influence the use of health services. We have also done considerable work in previous studies, specifically in continuing care seniors’ facilities, that expands knowledge of the influence of organizational contextual factors in health services [49,53-55]. The importance of studying the health services context is also well supported by others [56-58].

### Setting

This study is being conducted in Kelowna, British Columbia, and Edmonton, Alberta. These two cities and surrounding areas are different in size, population and health system composition. In 2006, Kelowna’s population was approximately 110,000 while Edmonton’s was just over 1 million. Kelowna has an older population compared to Edmonton (18% compared to 10.7%) [60]. The two regions are organized differently with regard to NH-ED transitions. In Kelowna, the General Hospital (KGH) is the sole receiving hospital for all 13 nursing homes in the Central Okanagan district and the only tertiary referral hospital in the Okanagan area, whereas in Edmonton, the University of Alberta Hospital (UAH) is one of five receiving hospitals within the city, one of 11 within the greater Edmonton region, and one of two tertiary referral hospitals. These factors offer an opportunity to study NH-ED transitions in two contrasting but adjacent provinces, enabling us to assess whether two contextually different systems may offer relative advantages or disadvantages to a successful transition experience. Both IH and AHS have a strong desire to build long term research capacity with committed researchers/decision-makers.

### Sample and inclusion criteria

All data collection will use purposive, convenience samples drawn from the populations as specified below.

1. **Nursing Homes**: The population consists of 50 NHs (13 in Central Okanagan and 37 in Edmonton). Forty-two of the 50 NHs send residents to these two EDs (all NHs in Central Okanagan and 29 of 37 in Edmonton). We will examine transfers from these 42 NHs and will do in-depth analyses on a sample of NHs using a sampling matric of high and low transfers (calculated as the number of annual transfers per number of beds per facility), and public, and private ownership. Eligible research participants include all individuals in the following groups – transferred NH residents and their families, physicians, registered and licensed practical nurses, healthcare aides and care managers.

2. **Emergency Medical Services**: All 88 EMS staff (6 full time and 6 part time ambulance crews) in Kelowna and the 556 EMS and 145 Inter-hospital Transport (IHT) staff in Edmonton will be eligible, as are the medical and administrative supervisors in each city.

3. **Emergency departments**: The physicians, nurses and other ED staff of the two EDs (KGH and the UAH) will be eligible for inclusion.

### Preparatory work for transition tracking - qualitative interviews & tool development

We received preliminary ethical and operational approvals to conduct the study in both provinces. Subsequent ethics amendments were approved once the data collection instruments were developed.

**Figure 4:**
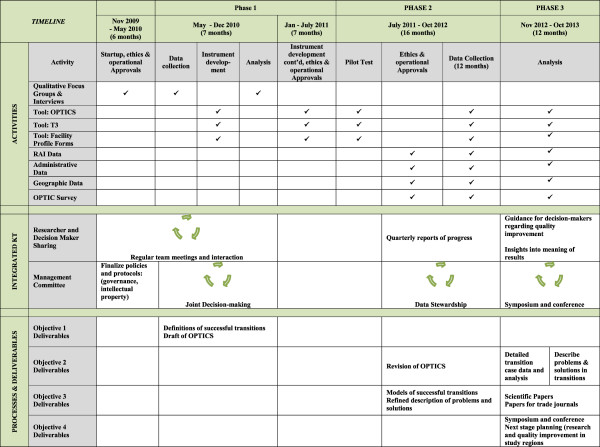
OPTIC TimeLine.

The study is being conducted in three phases; Phase 1 consisted of qualitative methods to investigate multiple perspectives of NH-ED care transitions and was completed to determine: (a) elements contributing to successful and unsuccessful transitions of care, and (b) measureable indicators for the initial Older Persons’ Transitions in Care Success (OPTICS) tool to track transitions in Phase 2. Thus the Phase 1 interviews informed the development of this protocol for Phases 2 and 3 (the focus of this manuscript). See Figure 4 for the OPTIC Study Timeline.

Phase 1 qualitative interviews allowed us to explore different perspectives of care transition experiences in our three settings. Semi-structured face-to-face interviews with 71 participants consisting of three groups of stakeholders (residents and families, frontline healthcare providers, and managers/administrators) in both provinces were employed to elicit key elements of success in all transition settings (NH, EMS and ED).

The findings of the qualitative interviews included five elements that contributed to the success of transitions and reflected a patient- and family-centered approach to care. Transitions were influenced by the complex interplay of multiple elements that included: knowing the resident; critical geriatric knowledge and skilled assessment; positive relationships; effective communication; and timeliness. When one or more of these elements was absent or compromised, the success of the transition was also compromised [59]. More information on the qualitative phase can be found elsewhere [59]. These elements and the IOM Model for Quality [51] led us to develop the OPTIC definition of successful transitions - *A successful transition is a coordinated set of actions that optimizes safety*, *resident centeredness*, *effectiveness*, *efficiency*, *timeliness and equity*, *across the entire transition*.

#### Development of the Transition Tracking Tool (T3)

The Transition Tracking Tool (T3) was developed from the OPTIC Transition Framework (Figure 1) and the findings from Phase 1 interviews [59]. It will be used to obtain case related transition data about individual NH residents beginning with the decision to transfer from the NH to the ED and ending with the return of the resident to the NH (understanding that some of these persons will die in hospital). The T3 consists of approximately 800 data points and incorporates the following categories of elements obtained by OPTIC staff from the resident/patient care records in each transition setting.

**NH**: Demographic and medical data (including medications), reason(s) for transfer, information about the decision and timing of transfer, accompanying documentation during resident transfer from EMS and ED, an assessment of handover communication between NH and EMS, and documentation of resident’s personal aides to daily living (such as eyeglasses, hearing aids, dentures);

**EMS**: The Canadian Triage Acuity Scale (CTAS) [44,45] was developed by the Canadian Association of Emergency Physicians (CAEP) and the National Emergency Nurses Association (NENA) and is used by EMS and EDs to prioritize patient care requirements, and to ensure that the sickest patients are seen on a priority basis when ED capacity has been exceeded due to high admission volume or restricted access to other services [45]. It has been shown to be valid and reliable, especially when applied by experienced nurses and using electronic decision support tools. Categories of data collected include CTAS scores, an assessment of documentation received from the nursing home that were prepared or received for ED use, timing of notification that a resident requires transfer (notification and actual transfer times, arrival at ED), an assessment of handover communication between the NH and EMS not captured in the documentation;

**ED**: Time of arrival, time of placement, time of ED physician assessment, time of consultation, time of disposition, investigations, diagnostic lists, reason for admission, overall length of ED stay (sub-divided into admitted and discharged patients);

**Disposition**: The location of the resident following transfer to the ED (inpatient, return to original NH, transfer to another NH, or death);

**Discharge from ED to EMS**: Adequacy of communication between the ED and EMS, accompanying documentation with the resident during transfer, timing of notification and actual transfer; and,

**Return to NH**: medical data from ED, accompanying documentation during resident transfer from EMS and ED, and assessment of communication at handover.

#### Development of the ‘Older Persons’ Transitions in Care Success’ (OPTICS) Tool (Outcome Measure #1)

For our work in Phase 2, we required a “transition quality outcome” variable to measure transition success. Using the criteria of (1) feasibility (brevity and ease of completion; the instrument can be completed in 10–15 minutes), and (2) modifiability (focus on concepts that are potentially modifiable), we developed the OPTICS tool to measure success of residents’ care transitions as perceived by the residents and by their family caregivers. The *OPTICS Scale for Residents* and the *OPTICS Scale for Family Caregivers* each consists of 14 questions related to the care received during the EMS portion (6 items based on the six quality domains in the IOM model and our definition of successful transitions), the ED portion (6 items) of the transition and two overall questions. To develop the two OPTICS scales, we engaged in an iterative process that involved the generation of initial items based on Phase 1 qualitative interview data, an assessment of face validity and feasibility, further item revision, and field testing prior to final item generation and assessment. With the services of Nooro Online Research (https://nooro.com), we developed an online program using the iPad (Apple Inc. http://www.apple.com/ca/ipad) for entry of transition tracking data.

During the final two months of Phase 1, we developed the processes for recruiting residents and their family caregivers into the study for each provincial study site, and for approaching the resident. Our approach to recruitment of residents differs depending on their level of cognition. Residents with a Cognitive Performance Scale score of 2 or less [60] who experience a transition will be approached by the Care Manager or designate in the NH to obtain verbal consent for a researcher to complete the OPTICS tool. If they agree to participate, informed written consent will be obtained by the OPTIC research staff. A family member whom the NH staff identify as being involved in the transition will also be approached and asked to provide consent to be interviewed about their own perceptions regarding the resident’s transition. Residents with a Cognitive Performance Score [60] 3 or more will not be approached to complete the OPTICS tool. In such cases, their family members whom the NH staff identify as being involved in the transition will be approached and asked to provide their own perspectives of the resident’s transition.

### Protocol for phases 2 and 3

#### Phase 2 – transition tracking

**Figure 5:**
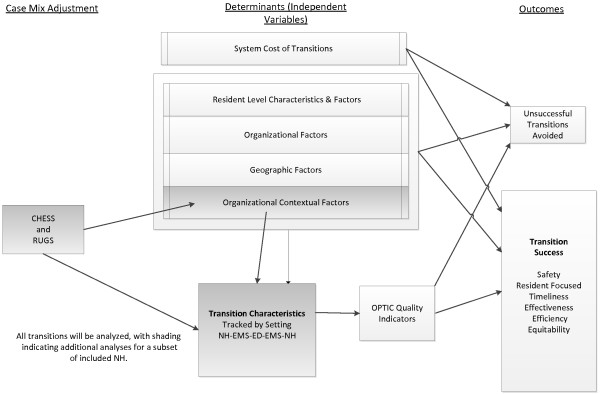
OPTIC Conceptual Model.

During Phase 2, the focus of this protocol, we will pilot test all of the data collection tools and then track approximately 400 transitions at the individual level using the T3 and OPTICS data collection instrument described above. We will also collect administrative data at the facility/organization, department and/or care unit levels for each of the settings in the study to allow us to analyze costs of transitions, to determine the relationship of organizational context and other characteristics such as workload to the successfulness of transitions. Each of these data measures and sources are described below and summarized in Table 1. The OPTIC Conceptual Model of the relationships among study concepts is presented in Figure 5.

**Table 1:** Data sources and measures

**Variable / Instrument**	**Source of data**	**Data type**	**Purpose**	**Unit of analysis**	**Collection phase**
**Individual Level Data**					
Semi Structured Interviews (Resident/family), and focus groups (providers)	Resident/family, healthcare providers, managers/ administrators in NH, EMS, ED	Qualitative	Identify perspectives on transitions and key indicators for initial development of OPTICS	Settings, residents, healthcare provider groups	Phase 1
Transition Tracking Tool (T3)	NH, EMS, ED	Mixed case specific	Obtain case-related data to track processes and events in transition	Individual	Phase 2
	Reason for transfer, timing, communication, results of transfer, disposition, priority, CTAS, etc.				
Perceptions of the transition process	Health care providers (nurses, paramedics)	Brief Survey	To obtain perspectives on need for the transition and quality of information sharing	Individual	Phase 2
Older Persons Transitions in Care Success (OPTICS)	Residents and their Family Caregivers	Mixed	Evaluate care transitions	Individual	Phase 2
**Facility Level Data**					
MDS-RAI 2.0 RUGS & CHESS	BC: Interior Health Region	Administrative	To adjust for case mix by NH	Facility	Phase 2
	AB: Individual Nursing Homes				
Demographic Profile Form	Facility and setting Managers in NH, EMS, ED	Semi Structured Interviews / Surveys	Constructing independent variable	Facility	Phase 2
OPTIC Survey Data	Health Care Aides in nursing homes	CAPI (computer assisted personal interviews)	To obtain a measure of context, and workforce characteristics such as job satisfaction, burnout etc.	Aggregated scores at Unit level	Phase 2
(Organizational context, burnout, job satisfaction, etc.)					

### Measures

#### Transition Tracking Tool (T3)

Individual level data from the first 50 consecutive residents experiencing a transition (911 call to EMS) across the two provinces will be assessed in a feasibility pilot to determine if and where data can be retrieved. We will also assess the timeliness of accessing patient records in each study setting to retrieve data. The piloted and revised T3 will then be used to track transitions for the one year of transition tracking. The sample will consist of all emergency (911) transitions from NHs in the Kelowna area to their ED (N=approximately 200) and all NH transitions from nursing homes enrolled in the Edmonton area to the study ED (N=approximately 200). We will recruit and have complete detailed case tracking for these approximately 400 cases from approximately 600 available cases (based on pre-study data) accounting for losses to attrition, inability to obtain consent, refusal to participate and incomplete records. We will attempt to recruit all available cases, even those who present during times when research staff are not immediately available. Transitions will be identified and accessed by the research team within three working days of occurrence so that detail missing from the records can be sought from healthcare providers.

#### Older Persons’ Transitions in Care Success (OPTICS) tool

The *OPTICS Scale for Residents* and the *OPTICS Scale for Family Caregivers* to assess transition successfulness will be pilot tested on the first 50 transitions and then revised as necessary through an expedited team meeting. The revised tool will be used for the one year of transition tracking along with the T3.

#### Organizational factors (Facility Profile Forms)

We will collect organizational data using a facility profile form for each NH, EMS, and ED. This will allow us to construct a comprehensive evaluation of current workload, staffing and transfer activity during the period of the study, to describe changes over time and to identify areas of highest priority for interventions. The sample includes facility management at all NHs, EMS and EDs. Data will be collected for the full year of transition tracking by requesting monthly service level data (e.g., ED visits, EMS calls and times, etc.) from each organization. We will also collect administrative data from EMS that describes transfer activity from all NHs to all EDs in for the three-year study period. This will allow us to compare our transition tracking activity to the volume of all NH-ED transfers in each region for the year before and after our transition tracking.

#### Economic data

We will measure direct costs at a variety of levels (health system, facility, and individual transition) and develop status quo cost per transition estimates for all NH-ED transitions using administrative data from the health authorities in each of the provinces. Detailed costing data such as personnel time, tests, procedures, and ambulance costs, will be obtained from the health authority administrative databases and time estimates will also be obtained from the T3. We will analyze the direct system costs of both successful and unsuccessful transitions and report differences. We will measure these status quo costs against our other health system outcome measure of interest - the direct cost per unsuccessful transition avoided.

#### Geographic data

We will collect postal codes of the NHs and the EDs for specific analysis of location and distance between facilities, on transition success. Transitions will be examined through a geographic lens to better characterize the role of places (NHs and EDs) in these events. This will capture movement of individuals through the health care system (i.e., as they move from place to place) and patterns in the variables related to that movement. Purposeful examination of this aggregated data will facilitate identification of strengths and gaps in transition patterns and contribute to hypothesis generation concerning attributes of successful (or not) transitions. Maps of transition patterns (and related variables including time, origin/destination attributes, transition volume, etc.) are useful visual aids to support communication with decision-makers and other key stakeholders. Further, ratings of transition success between institutions as measured by the OPTICS tool will be mapped relative to locations and both location and transition attributes.

#### Organizational context

After six months of transition tracking data have been collected, we will select a representative sample of 4–5 NHs in each province. We will identify in our sampling matrix of high, medium and low transfer facilities matched with large and small bed facilities, and public, private, not-for-profit ownership informed by the Translating Research in Elder Care (TREC) study [61,62]. These selected NHs will provide a total of 15 care units per province (average 3 units per facility), where we will collect data to measure organizational context (discussed in detail below). We will adjust for resident acuity in a NH using Resource Utilization Groups (RUGs) and Changes in Health, Endstage Disease and Symptoms and Signs (CHESS) scores.

#### RUGS & CHESS

The Minimum Data Set-Resident Assessment Instrument (MDS-RAI 2.0) is part of an international system intended to capture information about the health, physical, mental, and functional status of NH residents [63-71] and is routinely collected by all NHs in BC and AB. We will collect RUGs and CHESS scores for the 30 care units (15 per province) in our sampling matrix. The scores will be used to adjust for case mix at the NH unit level in our multivariate analytic models (described below). The Version III (RUGs III) system has been validated in multiple settings [72-74]. RUGs III is a case mix classification system developed to monitor, track, and benchmark staffing and resident resource use. Data have been used to guide local and organizational decision making in regards to resource use and allocation [72,75], which acts as a proxy for resident need. CHESS is comprised of MDS-RAI 2.0 data and is used to identify patients at risk for serious decline in health or mortality [76]. CHESS has predictive validity as it predicts mortality independent of age, sex, disability, cognitive performance, and Do Not Resuscitate orders [76]. Each increment on CHESS is associated with a distinct survival curve, with higher scores corresponding to a reduction in probability of survival. The sample will include the 15 units in the 4–5 participating NHs in each province where we will measure context.

#### Context measure

Healthcare aides (HCAs) in these selected facilities will be asked to complete the Alberta Context Tool (ACT) [49]. The ACT measures 10 contextual concepts: (1) leadership, (2) culture, (3) evaluation, (4) social capital, (5) structural and electronic resources, (6) formal interactions, (7) informal interactions, (8) organizational slack - staffing, (9) organizational slack - space, and (10) organizational slack – time [49], and is a validated and reliable instrument [49,62,77,78]. We will also collect data from healthcare aides on:

•Demographics including age, gender, education, job training, length of time working in their primary facility, and shift most frequently worked.

•Job Satisfaction (using a single item)

•Burnout (using the Maslach Burnout Inventory, Short form GS [79])

The sample will include 300 HCAs (10 HCAs per care unit for 15 care units per province) using convenience sampling on each unit. Previous studies utilizing the ACT have shown that 10 surveys per unit provide a stable measure of unit-level context [62,77,78]. The inclusion criteria for healthcare aides [80] to complete the survey are:

•Employed by their facility for at least 3 months;

•Work a minimum of 6 shifts per month;

•Able to identify a unit where they work most of the time.

### Outcome measures

There are four outcomes of interest in this study: the primary outcome of Resident and Family Caregiver perceptions of the successfulness of their transition (*OPTICS* described in Phase 1) and three secondary outcomes, described below.

#### Nurse and paramedic perceptions of quality (Outcome Measure #2)

We will use a short instrument developed for this study to obtain the perceptions of nurses in the ED, paramedics in EMS, and nurses in the NH after the resident returned. These questions will be asked for each transition and will address the quality of the handover information received, the quality of the interchange, and their perception of whether the transition could have been avoided and, if they believe that it could have been prevented, what could have prevented the transition.

#### OPTICS quality indicators (Outcome Measure #3)

An intermediate outcome measure is the *OPTICS Quality Indicators*, being developed for this study (see Figure 5). These are a series of quality indicators for each of the six domains of quality in the IOM model which are being derived from the growing research and guidelines literature on transitions generally [81], specific to NH-ED [27,82], and in specific settings like EDs [82,83]. For example, we know that communication of resident information between healthcare providers during handover of responsibility for the resident across health system sectors is an important safety indicator [84]. We will identify from the literature the key indicators of communication that act to support resident safety during transitions [85].

#### Unsuccessful transitions avoided (Outcome #4)

Through research team analysis of T3 and OPTICS data, we will identify unsuccessful transitions that should not have occurred. For example, our research team may determine that a transition was initiated when the difficulty experienced by the resident could have more appropriately been dealt with in the NH.

### Analyses

Planned analyses and deliverables are based on the OPTIC Conceptual Model (Figure 5).

### Psychometrics of the OPTICS tool

We will use the first 50 cases to undertake refinement of the OPTICS tool and will use the remaining cases to assess its psychometric properties (assessing internal consistency, item-total correlations, and dimensionality using exploratory factor analysis). We will have sufficient statistical power for psychometric assessment using exploratory factor analytic techniques with the remaining cases. We do not anticipate that we will have sufficient data to carry out a confirmatory factor analysis. In an iterative fashion, we will also carefully assess feasibility and practicality of the tool. In this phase we will also identify problems in the transition process and preliminary solutions to these problems. During this tracking phase, the RUGS and CHESS scores for the NHs from MDS-RAI 2.0 custodians will be collected. The data for geographic analysis will also be collected at this point.

### Modeling the factors related to transition success

We will construct models of association between contextual, economic, geographic, and resident indicators and transition success, to assess the relationships identified in the OPTIC model (Figure 5). We will build and analyze final models of association, for example, regression models with cluster correction for organizational unit, to determine which factors are significant predictors of transition success and unsuccessful transitions avoided. Using a random coefficient model, these types of equations will be of the following form: Yij = (a + ß.ij) + (vj.ij + μj+ eij), where Yij is the dependent variable for observation i in cluster j; a is the intercept; b is the effect of the covariate of .ij ; vj is the amount by which the coefficient of cluster j deviates from the average b; uj is the Level-2 random effect (clustered or group); eij is the Level-1 random effect (individual). We will construct a more refined description of the problems and potential solutions following careful assessment of these models, the process data we will collect throughout, and regular team meetings and discussions. In this way, we will investigate the role of context in the frequency, timing and type of transitions from NH to ED. Similar analytic techniques incorporating additional variables will be performed on a sub-set of cases (N= approx. 300 health care aides) to determine the effects of organizational context factors (ACT) on transition success when controlling for case mix (RUGS and CHESS).

#### Economic analyses

We will consider the economic impact of patient transitions to and from the ED by evaluating two costing scenarios – status quo and successful transition. Data on health system average costs related to transitions of NH residents from NH via EMS to EDs and back will be derived from the administrative data. We will analyze the costs of loss of residents’ aids to daily living both in average financial costs and potential consequences for the residents’ quality of life. We will also calculate the costs associated with unsuccessful transitions avoided. This will allow us to build a complete and accurate picture of transfer activity during the period of the study, to describe changes over time and to identify areas of highest priority for interventions.

#### Geographic analyses

Using a health geographic lens to map care transitions, we will also characterize the role of place in the transition process. Specifically, we will map locations of where transfers originated from and ended at, characteristics of origin and destination locations related to attributes of transitions (e.g., time, OPTICS score, etc.), and variations in time taken for transfers between destinations to elucidate overall patterns (e.g., from initial call to ambulance at origin to arrival at hospital and all noted time points in between) relative to those (measured) contextual factors that explain variation in overall transfer times.

#### Ethical research conduct and data management

Ethics approval involved three different forms of consent procedures, depending on the sample and data source. 1) For Phase 2 Transition Tracking, we received approval from the ethics board to waive written consent from each NH resident on the basis of the following:

1. It would not be reasonable to ask a NH resident who may have significant baseline cognitive deficits to provide consent to participate in a study if they were already in the process of a transition.

2. It would not be reasonable to ask all NH residents in the study cities (several thousands) to consent *a priori* to a study in the event that they may have a transition over the course of the following year.

3. Frail elderly NH residents who might potentially benefit from the conduct of health services research that could inform ways to improve the care they receive should not be excluded from such research.

4. Not all NH residents have a close family member/caregiver who had Power of Attorney to provide consent on their behalf.

5. Data collected using the T3 tool used resident/patient care records which would subsequently be de-identified.

Residents and family caregivers provided informed verbal consent to be interviewed in Phase 1 and for Phase 2 OPTICS questions. Healthcare providers provided informed verbal consent to be interviewed in Phase 1 and for Phase 2 perception questions.

This study is being conducted in accordance with the Tri-Council standards for research with vulnerable populations [86] and the Health Research Ethics Board guidelines at the involved Universities and health regions. All data handling adheres to data security policies of the Universities and Health Research Ethics Boards concerned. Data will be managed centrally in accordance with Tri-Council standards and stored in the secure data repository at the University of Alberta’s Faculty of Nursing. Appropriate access for University of British Columbia investigators, Interior Health and the Edmonton region decision-makers will be made in accordance with study specific data management and security arrangements governed by the OPTIC Data Management Committee. In this study it is not possible to anonymize the NH, ED, or the EMS teams. We have discussed this explicitly with all participating organizations.

### Return on investment: knowledge translation and dissemination

The return on this investment will take two major forms: integrated KT approaches and end-of-grant knowledge translation (KT). IKT involves regular team meetings and other forms of disciplined interaction, as well as joint decision-making through project management committees comprised of a balanced set of key researchers and decision-makers. If additional funds can be secured, we will host a Transitions symposium to results and implications for a wider audience of decision-makers and clinicians. KT will consist of peer review publications and conference presentations for research audiences and, for system administrators and managers, reports in trade journals and in relevant meetings and conferences.

## Discussion

Key to study success is operationalizing the IKT approach by using a partnership model in which the OPTIC governance structure provides for team decision-makers and researchers to participate equally in developing study goals, design, data collection, analysis and implications of findings. As preliminary and ongoing study findings are developed, their implications for practice and policy in study settings will be discussed by the research team and shared with study site administrators and staff. The study is designed to investigate the complexities of transitions and to enhance the potential for successful and sustained improvement of these transitions.

Study deliverables related to transition include definitions for successful and unsuccessful transitions from multiple perspectives, descriptions of potential problems and solutions to the management of transitions, development and testing of a feasible and practical tool to measure success of transitions, and diagnostics to support areas of strategic focus and development by decision-makers regarding seniors’ transitions. Advancements in processes for IKT will include (i) extended new KT practices between decision-makers and researchers, (ii) increased health system knowledge and awareness of issues, (iii) trained new scientists as a result of engaging research trainees, and (iv) continued development of professional relationships that will, result future collaborations.

## Abbreviations

ACT: Alberta Context Tool; AHS: Alberta Health Services; CIHR-PHSI: Canadian Institutes of Health Research, Partnerships for Health System Improvement; CPS: Cognitive Performance Score; CTAS: Canadian Triage Assessment Score; ED: Emergency Department; EMS: Emergency Medical Services; HCA: Healthcare Aides; IH: Interior Health Authority; IKT: Integrated knowledge translation; LTC: Long term Care; NH: Nursing Homes; OPTIC: Older Persons’ Transitions in Care; OPTICS: Older Persons’ Transitions in Care Success; T3: Transition Tracking Tool.

## Competing interests

The authors declare that they have no competing interests.

## Authors’ contributions

GGC is the nominated principal investigator for the OPTIC research program, is providing leadership and coordination for the program and is provincial lead for Alberta. RCR is a principal investigator, and provincial lead for British Columbia. CAE and PGN conceived of the original idea as complimentary to the TREC (Translating Research in Elder Care) program, and worked collaboratively with GGC and RCR to secure funding including partnership grants. GEC leads the Transitions Tracking Tool project (Phase 2) and is the EMS lead. BHR provides access to Emergency Department for data collection as the Alberta ED lead. JLB and CAR led the qualitative working group (Phase 1). JL contributed a Gerontological ED perspective. LB, SA, SL and EM contributed to detailing and operationalizing protocols for data collection. All authors read and approved the final submitted manuscript.

## Pre-publication history

The pre-publication history for this paper can be accessed here:

http://www.biomedcentral.com/1471-2318/12/75/prepub
